# Comparative analysis of hydroxyapatite and zinc oxide nanoparticles for effective dentinal tubule occlusion in dentin hypersensitivity management: a profilometric and scanning electron microscopic investigation

**DOI:** 10.1186/s12903-025-07063-2

**Published:** 2025-10-31

**Authors:** Mayar H. Hassaan, Nagah A. Rashad, Afaf A. El Sawa, Lubna M. Eldesouky, Aya S. Sedik

**Affiliations:** 1 Department of Oral Biology, College of Dentistry, Arab Academy for Science, Technology & Maritime Transport, El Alamein, Egypt; 2 Department of Oral Biology, Faculty of Dentistry, Alexandria University, Alexandria, Egypt; 3 Department of Pharmaceutics, Faculty of Pharmacy, Alexandria University, Alexandria, Egypt

**Keywords:** Dentin hypersensitivity, Nanoparticles, Hydroxyapatite nanoparticles, Zinc oxide nanoparticles, Carboxymethyl cellulose hydrogel, Dentin

## Abstract

**Background:**

The objective of this study was to evaluate the efficacy of hydroxyapatite nanoparticles (HANPs) in comparison to zinc oxide nanoparticles (ZnO NPs) in occluding dentinal tubules (DTs). Managing dentin hypersensitivity (DH) and assessing the resilience of anti-hypersensitivity treatments present significant clinical challenges. Hence, it is imperative to explore the impact of NPs on DH treatment.

**Methods:**

In this study, twenty- seven orthodontically extracted teeth with no caries or restorations were employed to produce 27 dentin discs. These discs were created by slicing the teeth coronally and subsequently subjecting them to a 20-second etching using 37% orthophosphoric acid to simulate DH conditions and eliminate the smear layer. The dentin discs were then randomly assigned to three groups: Group I (etched control), Group II (HANPs), where dentin discs were treated with carboxymethyl cellulose **(**CMC) dental hydrogel loaded with HANPs, and Group III (ZnO NPs), where dentin discs received treatment with CMC dental hydrogel loaded with ZnO NPs. Finally, the dentin discs underwent various analyses, including profilometric assessment to measure surface roughness (SRa) of dentin discs, qualitative assessment using scanning electron microscopy (SEM) to evaluate DT occlusion and quantitative assessment SEM images using Image J software platform.

**Results:**

Analysis of the dentin discs revealed that Group I had the highest SRa measuring 1.52 ± 0.08 μm, followed by Group II, measuring 1.21 ± 0.06 μm, while Group III exhibited the lowest SRa measuring 1.20 ± 0.05 μm. SEM examination indicated that Group III displayed the most extensive DT occlusion, followed by Group II, while Group I exhibited the lowest level of occlusion. The results from the SEM analysis were quantitatively validated through additional analysis using Image J software. Statistical analysis (ANOVA and post hoc Tukey’s test, *p* ≤ 0.05) revealed significant differences between groups, thereby rejecting the null hypothesis.

**Conclusions:**

The application of ZnO NPs demonstrates a positive impact on both the qualitative and quantitative aspects of DH.

## Highlights

• The inclusion of ZnO NPs is anticipated to enhance clinical outcome in reducing dentin hypersensitivity by occluding dentinal tubules

• All authors agree with the submission

• The work has not been published or submitted for publication elsewhere, either completely or in part, or in another form or language

• No conflict of interest with the authors in this work

## Background

Dentin hypersensitivity is highly prevalent on a global scale, and with a decline in the prevalence of other oral disorders, DH has become a prominent concern [1].

Dentin hypersensitivity often results from a combination of contributing factors. Changes in tooth structure, such as attrition, abrasion, and erosion, can lead to enamel loss. However, the most common cause of DH is believed to be gingival recession. Aggressive brushing, inadequate oral hygiene, and periodontitis can lead to the recession of the gingival margin, which, in turn, rapidly wears away the thin layer of cementum, exposing the underlying DTs [2].

To alleviate nerve sensitivity, potassium nitrate is included as an active component in toothpaste or mouthwash for DH treatment. Additionally, DT occlusion can be achieved through the application of substances like calcium hydroxide, fluorides, varnishes, and restorative materials [3].

Nevertheless, creating a durable and effective treatment for DH, which results in the formation of substantial remineralized layers capable of withstanding the mechanical and acidic challenges within the oral environment, poses a significant challenge [4].

Recent research has emphasized the importance and interest in biomimetic oral health products, leading to a growing focus on nanotechnology. This is primarily because nanotechnology offers unique characteristics like nano-sized particles, extensive surface area, and precision in targeting specific processes [5]. Hence, nanoparticles (NPs) have emerged as a promising solution for addressing DH [6]. The small size and increased reactivity of NPs provide a significant benefit in penetrating DTs, leading to improved potential for decontamination, remineralization, and reduced sensitivity when compared to traditional treatment approaches [7].

Previously, various NPs, including HA [8], Doxycycline-doped polymeric NPs and Ca-doped polymeric NPs have been investigated for DH treatment. Ca-doped NPs effectively occlude DTs by inducing Ca-P precipitates, significantly reducing fluid flow through dentin. However, they do not improve dentin’s mechanical properties or promote remodeling. On the other hand, doxycycline has dual functions as an antibacterial agent and a matrix metalloproteinase (MMP) inhibitor, but it leaves a significant portion of DTs unobstructed and does not reduce hydraulic conductance [1]. Furthermore, in the majority of instances, mineral precipitates are loosely formed and do not firmly attach to the matrix [9].

To address the drawbacks of existing agents, it is crucial to develop an occlusive agent that promotes the formation of a dense and durable mineralized layer within DTs, while being painless, user-friendly, and non-detrimental to dental and gingival health [8, 10].

Zinc oxide nanoparticles represent a category of metal oxide NPs with promising potential in various biomedical applications, including diagnostics and therapeutic interventions. The affordability, antibacterial characteristics, and overall safety of ZnO have garnered significant interest in the scientific community [11, 12].

Moreover, ZnO NPs have a wide range of applications within dentistry. These applications encompass enhancing the antibacterial attributes of traditional restorative materials, serving as desensitizing components in toothpaste, effectively combating harmful oral microorganisms in both antimicrobial and antifungal capacities, promoting the remineralization of dentinal lesions near cervical third, and improving the durability of localized drug delivery systems [12]. Furthermore, the presence of zinc in the metal oxide can help inhibit collagen degradation, promote the remineralization of dentin [13], and sealing of DTs [1].

In addition, ZnO NPs not only mechanically occlude DTs but also release Zn²⁺ ions that interact with phosphate ions in the dentinal fluid or saliva. This interaction leads to the in-situ formation of insoluble zinc-phosphate complexes, which firmly deposit within the tubules and on the dentin surface. This dual-action approach enhances both the immediate and long-term sealing efficacy of ZnO-based materials [1].

Studies have demonstrated that ZnO nanoparticles exhibit superior resistance to mechanical abrasion. When subjected to simulated toothbrushing, occlusion by ZnO NPs remained intact, with minimal loss of sealing material. This contrasts with conventional agents, which often experience partial or complete tubule reopening under the same conditions. The sustained presence of ZnO within DTs suggests a high potential for long-term clinical effectiveness [1].

The incorporation of ZnO nanoparticles in the treatment of DH has gained increasing attention due to their superior penetration into DTs and enhanced resistance to mechanical challenges. Nano-based desensitizing agents have been found to outperform traditional materials in terms of tubule occlusion depth and durability of the occlusive layer. Moreover, formulations containing ZnO nanoparticles have shown significant and sustained reductions in DH symptoms following clinical application, suggesting their potential for long-term effectiveness in managing the condition [1].

While HANPs have long been used for managing DH due to their biocompatibility and mineral-mimicking structure, recent research has highlighted the potential of ZnO NPs in achieving effective DT occlusion. In particular, ZnO NPs have demonstrated excellent retention within tubules after mechanical brushing, suggesting a promising degree of stability compared to other agents [9].

Despite promising results for both ZnO NPs and HANPs in previous studies, most investigations have examined these agents independently, often using varied methodologies that make direct comparison difficult. The present study is novel in that it directly compares HANPs and ZnO NPs under identical, standardised laboratory conditions, eliminating variability caused by differences in experimental design. This direct comparative approach allows a clearer evaluation of their relative DT occlusion potential and durability, providing evidence that is both clinically relevant and previously unavailable in the literature. The null hypothesis, based on the absence of prior direct comparative data and the assumption that both agents possess similar occlusive capabilities, posits that there would be no significant differences in DT occlusion or durability between the study groups under the applied testing conditions.

## Materials and methods

A total of twenty-seven dentin discs from 27 premolars, all of which had no carious lesions or restorations and were originally extracted for orthodontic reasons. These premolars were obtained from the Oral and Maxillofacial Surgery Department within the Faculty of Dentistry at Alexandria University. The study was carried out with the approval of the Ethical Committee at the Faculty of Dentistry, Alexandria University, under the reference numbers IRB NO: 00010556 and IORG 0008839.

This study is an in vitro comparative study. These twenty- seven premolars were sliced from their mid-coronal portions to create twenty- seven dentin discs. The randomization scheme used to allocate the twenty- seven dentin discs employed the permuted block technique [14], that was generated by using the website (http://www.Randomization.com). The dentin discs were randomly divided into 3 equal groups, 9 discs per group.

Group I: (*n* = 9) Etched control group.

Group II: (*n* = 9) Hydroxyapatite nanoparticles group.

Group III: (*n* = 9) Zinc oxide nanoparticles group.

Dentin disc preparations and toothbrushing by the custom-made Brushing Simulator, were carried out at the Biomaterials Department, Faculty of Dentistry, Alexandria University, Alexandria, Egypt. Material preparation took place at the Department of Pharmaceutics, Faculty of Pharmacy, Alexandria University, Alexandria, Egypt. The 3D Laser Scanning Microscope was utilized at Egypt-Japan University of Science and Technology, New Borg El-Arab City, Egypt. The transmission electron microscopy (TEM) Unit and SEM Unit were conducted at the Faculty of Science, Alexandria University, Alexandria, Egypt.

### Dentin disc preparation

The dentin disc preparation involved using a Microtome (Micracut 150, Metkon^®^ metallography, Turkey) to remove 2.5 mm of occlusal enamel from the cusp tip. This process yielded discs with a thickness of 2.0 mm (± 0.2 mm) obtained from the mid-coronal part by making a horizontal cut at the cemento-enamel junction of each tooth, extending from mesial to distal [9, 15].

### Simulating dentin hypersensitivity

The dentin discs obtained underwent a 20-second etching process using 37% orthophosphoric acid to open the DTs, creating a DH model [9], and simultaneously eliminating the smear layer [16]. Subsequently, they were thoroughly rinsed with distilled water for one minute [9].

### Preparation of artificial saliva [17]

The artificial saliva utilized in this study was formulated following Macknight-Hane and Whitford (1992). The composition of the synthetic saliva (in grams per liter) consisted of 2.00 g Methyl-p-hydroxybenzoate, 10.00 g Sodium CMC, 0.625 g KCl, 0.059 g MgCl_2_. 6H_2_O, 0.166 g CaCl_2_. 2 H2O, 0.804 g K2HPO_4_, and 0.326 g KH2PO_4_.

###  Orthophosphoric acid preparation (37%) [9]

To precisely create a 25 mL solution of 37% orthophosphoric acid, we combined 9.25 mL of orthophosphoric acid with water in a 25 mL volumetric flask, incrementally adding water until reaching the 25 mL mark. We then utilized a Sonicator to ensure complete mixing of the solution.

### Preparation of carboxymethyl cellulose dental hydrogel scaffold [8]

The CMC dental hydrogel scaffold was formulated by combining 0.8 g of CMC, 4 g of Glycerol, and 11.2 g of H2O in a beaker. The mixture was meticulously stirred using a magnetic stirrer until thoroughly blended.

### Preparation of carboxymethyl cellulose dental hydrogel scaffold loaded with hydroxyapatite nanoparticles [8]

To achieve a consistent blend, 4 g of HANPs (purchased from Nanotech, Cairo, Egypt) were introduced into the beaker containing the CMC dental hydrogel scaffold. The mixture was continuously stirred with a magnetic stirrer for one hour until it transformed into a paste with uniformity.

### Preparation of carboxymethyl cellulose dental hydrogel scaffold loaded with 20% zinc oxide nanoparticles [8]

To create the CMC dental hydrogel scaffold infused with 20% ZnO NPs, 4 g of ZnO NPs (purchased from Nanotech, Cairo, Egypt) were introduced into the beaker containing the CMC dental hydrogel scaffold. Continuous mixing with a magnetic stirrer was maintained for one hour until a uniform, paste-like consistency was attained.

### Etching procedure

The dentin discs underwent a 20-second etching process with 37% orthophosphoric acid to initiate the opening of DTs, thus establishing a DH model [9] and eliminating the smear layer [16], closely replicating the initial stages of dentin hypersensitivity as seen with acidic dietary challenges. Subsequently, the dentin discs were rinsed with distilled water for 1 min and subjected to 15 min of ultrasonic cleaning [9]. Following this, the discs were placed in artificial saliva to replicate oral conditions [18].

### Protocol for simulated toothbrushing of dentin discs [8]

The etched dentin discs were labeled on the non-brushing side with a corrector. They were affixed to a rubber base for stability during the brushing procedure. Dentin discs in groups II and III received applications of CMC dental hydrogel scaffold loaded with HANPs and CMC dental hydrogel scaffold loaded with ZnO NPs, respectively. Artificial saliva was introduced to simulate oral conditions during the brushing cycles, which mimics the ionic content and hydration of the natural oral environment. Using a custom-made Brushing Simulator (from the Dental Biomaterials Department, Faculty of Dentistry, Alexandria University), with the medium bristle stiffness and standard hand pressure (approx. 200 g), the brushing cycles were conducted for 2 min, three times a day to simulate realistic oral hygiene practices, accelerate wear for time-efficient testing, enhance material interaction, and ensure consistency with previous studies, over a span of 7 days, replicating the physical wear encountered during daily oral hygiene. Following brushing, the discs were rinsed with deionized water for 1 min and then returned to their respective containers filled with artificial saliva.

### Methods of specimens’ examination

#### Characterizing nanoparticle suspension using transmission electron microscopy [19]

Characterization of the NP suspension involved the use of a TEM with a 120 kV accelerating voltage (JEM-1400 series, USA). To observe the size and shape of the NPs, 100 mg of NPs were suspended in 200 mg of ethanol and subjected to 10 min of sonication. A small droplet of the NPs suspension was then placed onto TEM grids coated with a thin carbon film and allowed to evaporate. Electron micrographs were examined from various locations on the grid.

#### Profilometric assessment of dentin discs utilizing a 3D laser scanning microscope [20]

To obtain surface topography data, a 3D laser scanning microscope (Keyence VK-X100, Keyence GmbH, Neu-Isenbuerg, Germany) was utilized for measuring the average surface roughness (SRa) of the dentin discs. All measurements were conducted at a 20X magnification level using laser light microscopy with a laser wavelength of 658 nm. The specimens were positioned on a designated platform, and three distinct areas were measured on each specimen. The scanned area size was 705 × 705 μm, and the scanning speed was set at 102 μm/s. The roughness value (expressed in µm) was computed by averaging the values obtained from three different positions on each specimen. Additionally, visual images of the surfaces were captured for a qualitative assessment of SRa. These images were interpreted using a color scale and graphics, where each color represented a distinct value; negative values indicated pits, while positive values indicated peaks.

#### Scanning electron microscope evaluation of dentin discs [8, 21]

Dentin discs underwent a cleansing process with distilled water. Subsequently, they were allowed to air dry at room temperature and then further dehydrated in a freeze dryer. The dentin discs were mounted onto an aluminum stub using double-sided carbon tape. To prevent the accumulation of electrostatic charge, a very thin layer of gold was applied to the mounted dentin discs using a sputter coater. The layer was thin enough to avoid masking the surface layer and compromising resolution. Following these steps, the dentin discs were qualitatively assessed for the extent of DT occlusion using the Japan Electron Optics Laboratory Scanning Electron Microscope (JEOL SEM) at 5000x magnification.

#### Computer-assisted scanning electron microscopic analysis of dentin discs [22, 23]

To ensure a standardized quantitative assessment of the extent of dentin tubule (DT) occlusion, image analysis was conducted using the ImageJ software platform (version 1.53k, NIH, Bethesda, MD, USA). This analysis utilized a five-point scale based on the tubule occlusion classification scoring system previously established by Hemalatha Doppalapudi [23]. Given that this scoring system is widely used in contemporary research studies, it is regarded as the standard method in the field.

- Score 1—Occluded (100% of the tubules closed).
- Score 2—Mostly occluded (50% to less than 100% of the tubules closed).
- Score 3—Partially occluded (25% to less than 50% of the tubules closed).
- Score 4—Mostly unoccluded (less than 25% of the tubules closed).
- Score 5—Unoccluded (0%, no tubular closure observed).

#### Statistical analysis

The data obtained from Profilometric analysis were compiled and subjected to statistical evaluation. One Way ANOVA was performed to analyzed differences in surface roughness between groups, followed by Tukey’s post hoc test with Bonferroni correction. All tests were two tailed and significance level was set at p value ≤ 0.05. Data were analyzed using SPSS version 23, Armonk, NY, USA.

#### Randomization and blinding technique

A permuted block randomization sequence was generated using an online tool (http://www.randomization.com) to allocate 27 dentin discs equally into three groups (*n* = 9 per group). Allocation concealment was maintained using sealed opaque envelopes and each disc was assigned a coded identifier to ensure unbiased assignment. The examiner who performed profilometric measurements, SEM imaging, and quantitative ImageJ analysis was blinded to group allocation during all primary assessments. To ensure measurement reproducibility, intra-examiner reliability was assessed: the same blinded examiner re-evaluated 20% of randomly selected specimens for each technique after a two-week interval to minimize recall bias. Agreement between measurements was quantified using the intraclass correlation coefficient (ICC) calculated with a two-way mixed-effects model for absolute agreement; ICC values exceeded 0.80 for both SEM and profilometry, indicating excellent reproducibility.

## Results

### Characterization of nanoparticles

#### Zinc oxide nanoparticles

**Fig. 1:**
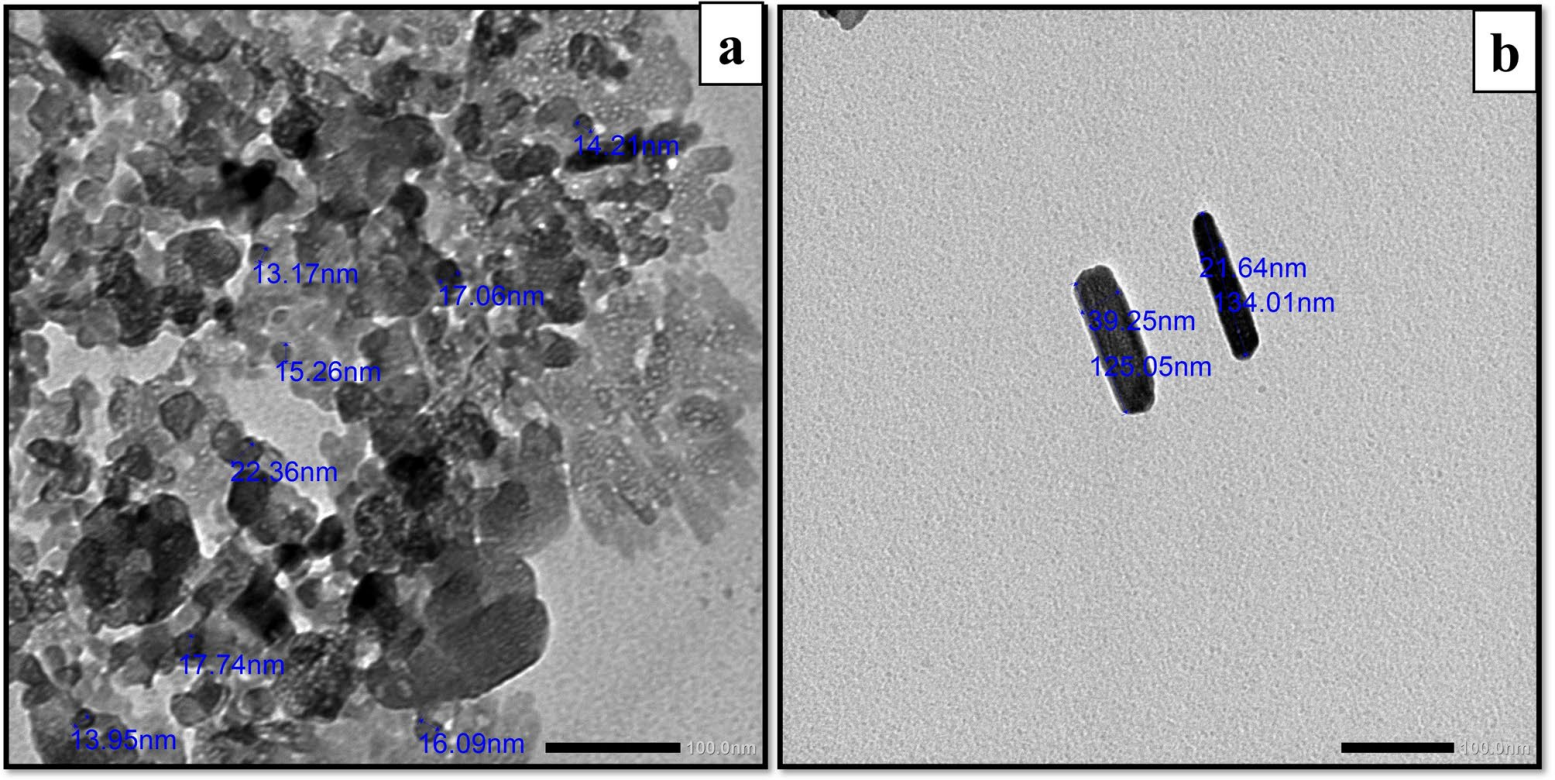
Characterization of nanoparticles by TEM. TEM image of ZnO NPs reveal the spherical-like morphology of the nanoparticles with sizes ranging from 13 to 22 nm (Scale bar; 100 nm) (**a**). TEM image of HANPs reveal the rod-like morphology of the nanoparticles with diameter ranging from 21 to 39 nm and length ranging 125 to 134 nm (Scale bar; 100 nm) (**b**)

Transmission Electron Microscopy analysis was employed to verify the size and shape of ZnO NPs, confirming their spherical morphology with dimensions averaging 30 ± 10 nm **(**Fig. 1a**).** The suspension of NPs was prepared by dispersing 100 mg of ZnO NPs in 200 mg of ethanol, followed by sonication for 10 min.

#### Hydroxyapatite nanoparticles

Hydroxyapatite nanoparticles displayed rod-shaped structures, with dimensions confirming their proper morphology: lengths ranged from 100 ± 30 nm, and diameters measured 25 ± 5 nm **(**Fig. 1b**).**

#### Profilometric analysis results

There was a statistically significant difference in SRa between the study groups, with p-value of ≤ 0.05.

**Fig. 2:**
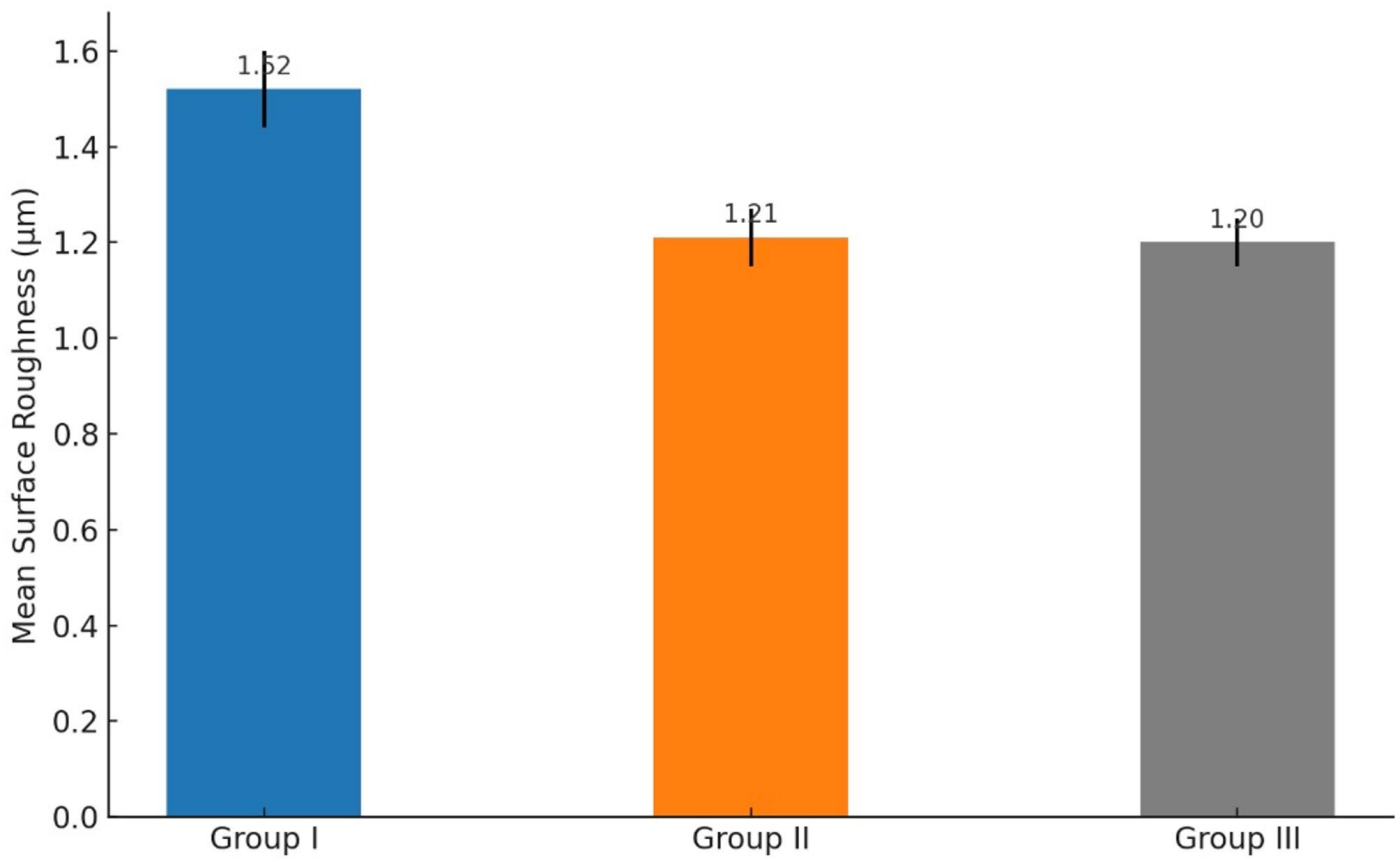
Comparison of surface roughness among the study groups, presented as a bar chart

Group I, the etched control group, showed the highest SRa, with a mean value of 1.52 μm, (95% CI: ±0.08). Group II, the HANPs group, displayed a mean value of 1.21 μm (95% CI: ±0.06), while Group III, the ZnO NPs group, showed the lowest SRa, with a mean value of 1.20 μm (95% CI: ±0.05), which were summarized in a bar graph **(**Fig. 2**).**

**Fig. 3:**
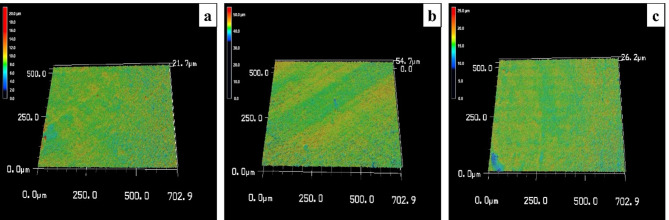
Profilometric analysis. The profilometric image of Group I (etched control group) shows the highest SRa among all groups, with an average mean value of 1.52 μm. In this image, blue represents the pits, while red indicates the peaks on the dentin discs (**a**). The profilometric image of Group II (HANPs group) shows a reduction in the SRa of the dentin discs, with an average mean value of 1.21 μm. This image also reveals fewer red peaks following brushing with HANPs (**b**). The profilometric image of Group III (ZnO NPs group) shows a reduction in the SRa of the dentin discs, with an average mean value of 1.2 μm. This image also reveals fewer red peaks following brushing with ZnO NPs (**c**)

Profilometric images depicting SRa across different groups revealed the following observations: In Group I, the image displayed the highest SRa, with blue indicating pits and red representing peaks **(**Fig. 3a**)**. Group II showed a decrease in SRa of dentin discs after brushing with HANPs **(**Fig. 3b**)**. Group III also exhibited a reduction in SRa of dentin discs after brushing with ZnO NPs **(**Fig. 3c**).**

#### Scanning electron micrograph results

SEM images of dentin discs from all study groups were chosen and captured at magnifications of 5000× to assess their surface characteristics and how effectively the NPs sealed the DTs.

**Fig. 4:**
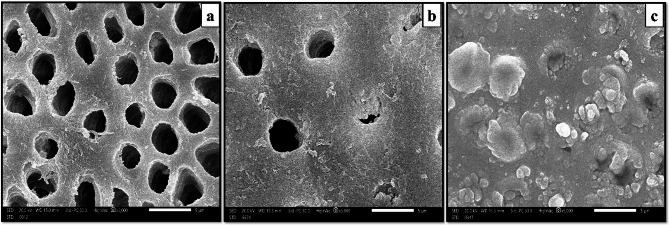
Scanning electron micrograph. SEM (Group I) shows a demineralized dentin surface with open DTs, and collapsed collagen network, resembling the characteristics of hypersensitive dentin. (x5000) (**a**). SEM (Group II) displays DTs sealed by HANP plugs and mineral deposits, with only a few tubules still open. (x5000) (**b**). SEM (Group III) reveals superior tubular occlusion due to retained ZnO NPs within the DTs. Spherical-shaped ZnO NPs effectively blocked the majority of the DTs. Some DTs remained partially open, with crystal formations or mineral deposits surrounding their openings (**c**)

Group I (etched control): dentin discs displayed a demineralized dentin surface with open DTs, and collapsed collagen network, resembling the characteristics of hypersensitive dentin **(**Fig. 4a**).**

Group II (HANPs): dentin discs displayed sealed DTs by HANP plugs and mineral deposits, with only a few tubules still open **(**Fig. 4b**).**

Group III (ZnO NPs): dentin discs exhibited superior tubular occlusion due to retained ZnO NPs within the DTs. Spherical-shaped ZnO NPs effectively blocked the majority of the DTs. Some DTs remained partially open, with crystal formations or mineral deposits surrounding their openings and in the peritubular and intertubular dentin. **(**Fig. 4c**)**

#### Computer-assisted scanning electron microscopic analysis results

Different quantitative analysis of the DTs occlusion was found in the various groups, according to Image J.

According to the percentages of the degree of DTs occlusion, which were summarized in the bar graph, (the 3 study groups (Group I, Group II, Group III) were compared [22].

**Fig. 5:**
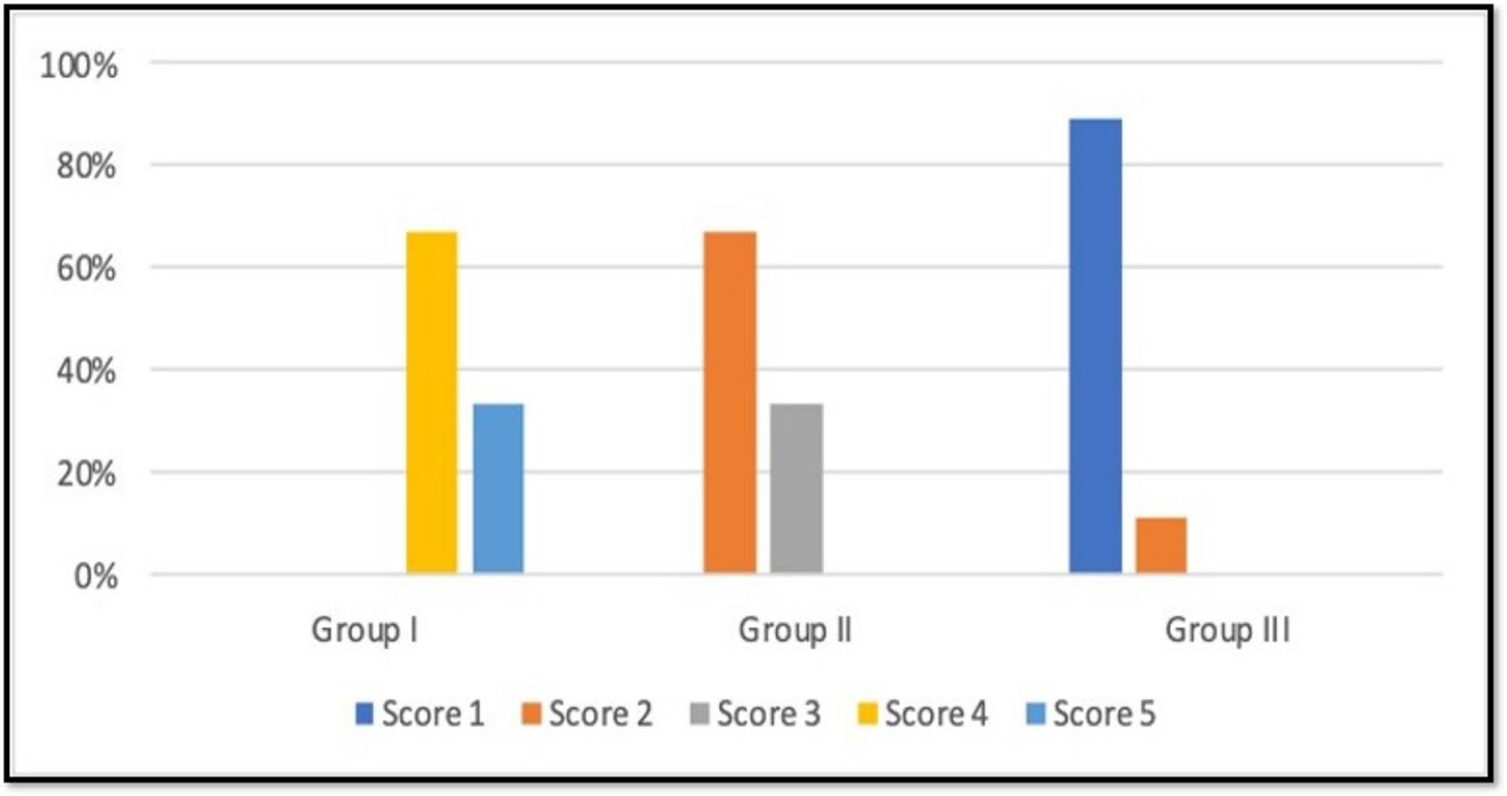
Quantitative assessment of the extent of dentinal tubule occlusion across the study groups, illustrated through a bar chart

Group I (etched control group) showed 66.7% of the dentin discs scored 4, while 33.3% scored 5. Group II (HANPs group) showed 66.7% of dentin discs scoring 2, while 33.3% scoring 3. Group III (ZnO NPs group) revealed that 88.9% of dentin discs scored 1, while only 11.1% scored 2. There was statistically significant difference between the study groups with p value ≤ 0.05. **(**Fig. 5**)**.

## Discussion

This study aimed to assess the efficacy of HANPs compared to ZnO NPs in occluding DTs. To achieve this, various analytical methods were employed, including TEM for characterization, 3D laser scanning microscope for profilometric analysis of dentin discs, SEM analysis of dentin discs, and computer-assisted SEM evaluation.

The study established a DH model using 2 mm thick dentin discs from extracted premolars. These discs were treated with 37% orthophosphoric acid for 20 s to expose DTs and replicate DH conditions [18]. The methodology aligns with the approach described by Khan et al. (2020), which also used it to create DH and assess the occlusion efficacy of experimental dentifrices [24].

In clinical practice, phosphoric acid is typically used at concentrations ranging from 30% to 40% [16]. Thus, in this study, a 37% orthophosphoric acid solution was utilized to remove the smear layer and replicate a DH model. Dentin discs were immersed in artificial saliva between brushing cycles to mimic the oral environment, in accordance with the methodology reported by Goldstein et al. (2017). This approach helps maintain a realistic oral environment during the study [18].

The findings of HANPs characterization align with the research conducted by Wang D et al. in 2016, which also confirmed the presence of rod-shaped HANPs with dimensions of 20 nm in width and 100 nm in length [25].

The results of ZnO NPs characterization align with Yusefi-Tanha E et al.‘s 2021 investigation on the effects of ZnO NPs, which assessed antioxidant defense biomarkers in relation to particle size, morphology, and concentration, confirming spherical particles with an average size of 38 nm [26].

The study results rejected the null hypothesis, demonstrating that the use of a CMC dental hydrogel scaffold loaded with ZnO NPs significantly enhanced DT occlusion and compared to the HANP loaded formulation. These outcomes highlight not only the therapeutic potential of ZnO NPs but also the functional role of the CMC hydrogel as an effective carrier system, facilitating NP retention, controlled release, and interaction with the dentin surface.

In this study, both the CMC dental hydrogel scaffolds containing HANPs and ZnO NPs exhibited the ability to occlude DTs. However, the ZnO NPs outperformed the HANPs, as evidenced by both qualitative assessments, such as DT occlusion observed in SEM images, and quantitative evaluations, including computer analysis using Image J, which confirmed the highest DT occlusion percentages. Profilometric analysis further supported these findings, revealing reduced SRa after treating with the CMC dental hydrogel scaffold loaded with ZnO NPs. This underscores the potential of ZnO NPs as a promising approach for addressing DH.

The profilometric analysis of Group I (etched control) revealed the most significant SRa (1.52 μm, 95% CI: ±0.08), confirming the success of the etching procedure in creating a DH model (*p* < 0.05). This aligns with Farooq et al. (2021) [27], who exposed dental blocks to citric acid, measuring SRa before and after demineralization, affirming that demineralization leads to increased SRa.

Profilometric analysis of Group II (HANPs group) demonstrated a decrease in dentin disc SRa after brushing with HANPs measuring 1.21 μm (95% CI: ±0.06), indicating that HANP application led to a smoothing effect on the dentin surface and reduced surface irregularities. Similarly, Group III (ZnO NPs group) exhibited the lowest SRa of 1.20 μm (95% CI: ±0.05), following treatment with the CMC dental hydrogel scaffold containing ZnO NPs, resulting in a smoother surface texture and confirming dentin remineralization.

This supports the findings of Farooq I et al. (2021) [27], who investigated the efficacy of a new toothpaste for remineralization. They assessed surface roughness and noted a decrease after treatment, indicating successful remineralization and smoother tooth surfaces.

The SEM examination of Group I (etched control) illustrated patent DTs and showed signs of dentin degradation along with collapsed collagen structure. This confirms the successful demineralization process and replication of the DH model. Furthermore, it affirms the thorough removal of the smear layer and plugs.

The results are consistent with a study by AS Khan et al. (2020) [9], who explored the DT occlusion capabilities of a newly developed dentifrice. In their research, dentin discs were etched with 37% orthophosphoric acid to simulate a DH model. Their findings showed that the group treated with artificial saliva did not exhibit significant DT occlusion which supports our current observations, highlighting that artificial saliva may not be an effective agent for DT occlusion in DH models.

Additionally, this study confirmed the successful removal of the smear layer, aligning with findings from a previous investigation by K Kripal et al. (2019) [28], who examined the effectiveness of propolis varnish in occluding DTs on dentin discs that were initially treated with 37% orthophosphoric acid to remove the smear layer and concluded that the acid etching process played a crucial role in exposing the DTs, ensuring they were free from any interference caused by the smear layer. This step was essential for accurately evaluating DT occlusion.

To strengthen our SEM findings and overcome limitations noted in Khan et al. (2020) [9], we conducted a quantitative analysis. Their study relied on manual counting of occluded DTs, a method susceptible to human error and reduced accuracy. To improve precision, a computer-assisted SEM analysis was conducted using ImageJ software.

Quantitative analysis of SEM images of Group I (etched control), confirmed the initial SEM findings. The results showed that 66.7% of the dentin discs received a score of 4, indicating that less than 25% of the tubules were closed. Additionally, 33.3% of the samples received a score of 5, representing 0% tubular closure with no observable closure of tubules. This quantitative data substantiates that a significant portion of the DTs remained open, consistent with the characteristics associated with DH. This is further supported by Gergely et al. (2010) [29], who presented additional evidence showing that DH regions display less than 25% DT closure when analyzed using SEM. Moreover, this aligns with Ashraf and Aidaros (2021) [21], who evaluated the effectiveness and durability of nano seashell, NaF, and commercially available toothpaste in treating DH and observed the lowest percentage of occluded DTs in the untreated control group.

The SEM examination of Group II (HANPs group) indicated that more than 50% of the DTs exhibited partial occlusion, with some tubules retaining HANP plugs. Additionally, mineral deposits were observed within the DTs, contributing to their occlusion. This demonstrates that HANPs was able to partially seal DTs, although the degree of occlusion was lower compared to Group III.

Moreover, this is consistent with the findings of Swarup and Rao (2012) [30], who conducted an assessment of the impact of synthetically produced HANPs on remineralization, comparing their effectiveness to sodium fluoride treatment and revealed that NPs with a diameter of 20 nm demonstrate a strong affinity for demineralized surface. Additionally, this aligns with Al-Maliky et al. (2014) [31], who explored an innovative treatment method that combined carbon dioxide laser therapy with a paste containing HANPs to occlude DTs and concluded that the NPs function as biologically active templates, effectively attracting significant quantities of calcium and phosphate ions from the remineralization solution, which in turn aids in the closure of the DTs.

This is consistent with the research conducted by Sadiasa et al. (2013) [8], who investigated the efficacy of a CMC dental hydrogel containing HA for addressing DH. Their approach involved the use of citric acid to etch dentin discs, followed by treatment with the specially formulated hydrogel and observed that the dentin disc surfaces exhibited irregular layers of crystalline-like deposits, effectively sealing the exposed DTs after 7 days of treatment. The inclusion of HA in the hydrogel provided an additional source of Ca and P ions during the mineralization process, contributing to the closure of the DTs.

A quantitative analysis of SEM images for Group II (HANPs group) supported the SEM observations, revealing that 66.7% of the dentin discs achieved a score of 2, indicating “closure of 50% to less than 100% of the tubules.” Meanwhile, 33.3% of the samples received a score of 3, signifying “closure of 25% to less than 50% of the tubules.

The SEM analysis of Group III (ZnO NPs group) showed enhanced DT occlusion attributed to the retention of ZnO NPs within these tubules. The spherical ZnO NPs effectively blocked the majority of DTs by penetrating and sealing their openings, mimicking the natural protective smear layer on dentin. Nonetheless, some DTs remained partially open, showing the development of crystalline structures or mineral deposits near their orifices and within the surrounding peritubular and intertubular dentin.

Our results are consistent with a study by Khan et al. in 2020, which showed that dentifrices containing ZnO NPs achieved the highest degree of DT occlusion, with more than 50% and less than 100% of the DTs being tightly sealed. This supports the effectiveness of ZnO NPs in occluding DTs [9].

Likewise, this supports the outcomes made by Toledano-Osorio et al. (2018) [1], in which Zn NPs demonstrated the highest rate of complete obliteration of DTs which was achieved by the formation of Ca-P precipitates, resulting in a substantial reduction in fluid flow within the dentin.

Furthermore, the findings align with Toledano et al. (2019) [32], who investigated four experimental NP solutions for reducing dentin permeability and observed a significant decrease in flow rate, attributed to a higher percentage of completely sealed DTs (100%) when using ZnO NPs, particularly in nanogels. Signifying its potential as a valuable therapy for addressing DH. Moreover, they verified the presence of strong dentin surface remineralization and the distinct periodic banding of collagen fibrils in cervical dentin following treatment with ZnNPs. Additionally, due to its smaller ionic radius compared to Ca, Zn can replace Ca in HA. Moreover, Zn helps maintain surface porosity, which aids in mineral entry [33]. In addition, they concluded that the biological apatite lacks Ca and has high carbonate concentrations. Although carbonated apatite is a precursor to HA, it forms a substituted apatite compound when precipitated in the presence of Zn due to an in vitro exchange between Zn^2+^ and Ca^2+^. Additionally, the possibility of intrafibrillar remineralization at partly demineralized collagen matrices is increased by the addition of Zn into NPs.

Further to this, our findings are supported by Khan et al. (2020) [9], who conducted a comparison of the in vitro potential for DT occlusion between two innovative experimental dentifrices, incorporating fluoride with bioactive glass and ZnO NPs. They concluded that these experimental dentifrices, which include ZnO NPs powders, offer an additional advantage. The incorporation of ZnO powder in toothpaste acts as a preservative, and when suspended in water, it not only prevents dentin demineralization but also elicits an antimicrobial effect by releasing Zn2 + and reactive oxygen species (ROS).

Moreover, this aligns with Toledano et al. (2020) [34], who conducted research to assess the efficacy of novel polymeric NPs in reducing dentin permeability and promoting dentin remineralization following endodontic treatment. Their study examined the impact of different NPs, including undoped NPs, Zn, Ca, and doxycycline-doped NPs on radicular dentin and discovered that the dentin treated with ZnO NPs exhibited increased concentrations of PG, which serve as adhesives between the collagen network and HA crystals. Additionally, they facilitate the release of small leucine-rich proteoglycans and small integrin-binding ligand N-linked glycoproteins from dentin via the activity of MMPs-3. These proteins participate in the mineralization of dentin, and the phosphorylated proteins that are immobilized cause the mineral to form. In partly demineralized dentin, ZnNPs also inhibit MMPs-3, which lowers collagen breakdown and encourages dentin re-mineralization.

The superior DT occlusion observed with ZnO NPs may be attributed, in part, to their nanoscale spherical morphology, which facilitates deeper and more uniform penetration into the tubule lumen compared to the irregular, rod-like structure often seen in HANPs. The smaller particle size of ZnO NPs (~ 20–50 nm) allows for enhanced packing density within the tubules, creating a more continuous and acid-resistant occlusive layer [9]. In contrast, HANPs, while biocompatible and chemically similar to natural apatite, may present size and shape limitations that reduce their ability to completely infiltrate and seal tubules, especially those with constricted diameters.

The SEM results of Group III (ZnO NPs group) were further validated through quantitative analysis, showing that 88.9% of the dentin discs received a score of 1, indicating complete closure of 100% of the DTs, while only 11.1% were given a score of 2, suggesting closure of 50% to less than 100% of the DTs. These results underscore that this group achieved the highest closure rates, affirming the effectiveness of ZnO NPs in sealing DTs and relieving DH.

Numerous studies have demonstrated the role of NPs in DT occlusion. Khan et al. (2020) [9], reported that ZnO NPs incorporated in dentifrices resulted in over 50% tubule occlusion following brushing, while Toledano-Osorio et al. (2021) [35], confirmed complete occlusion with Zn-doped nanogels under SEM imaging.

In summary, the thorough examination of profilometric measurements, scanning electron micrograph analysis and quantitative assessment carried out in this study provides compelling evidence for the viability of employing a CMC dental hydrogel scaffold infused with ZnO NPs as a means to shield the dental pulp from DT exposure. Additionally, it indicates that this strategy holds promise for mitigating or eradicating DH.

To further assess the clinical viability of ZnO NPs, it is essential to consider their potential cytotoxic effects and long-term safety. Although ZnO NPs have demonstrated antimicrobial and remineralization benefits, their biological safety must be established. Previous research by Sirelkhatim et al. (2015) [36] and Moradpoor et al. (2021) [37] has shown that ZnO NPs can be cytotoxic at high concentrations, particularly due to ROS generation. However, at low concentrations, they tend to exhibit acceptable biocompatibility, especially when incorporated within controlled delivery systems such as hydrogels.

This study has several limitations. The small sample size (*n* = 27) restricts generalizability, and the in vitro model does not fully replicate the oral environment, lacking factors such as saliva, enzymes, and microbes. Using only phosphoric acid etching may not accurately simulate clinical dentin hypersensitivity, where erosive and abrasive conditions are more representative. The durability of tubule occlusion over time was not assessed, and neither were the cytotoxicity and long-term biocompatibility of ZnO NPs within the CMC scaffold. EDX analysis was also not performed to confirm the chemical composition of the occluded deposits. Future research should include advanced material characterization and in vivo models incorporating erosive or abrasive cycling to better evaluate safety, effectiveness, and clinical relevance.

## Conclusions

The use of a dental hydrogel scaffold containing ZnO NPs demonstrated effectiveness in occluding DTs under in vitro conditions. The findings suggest that these NPs can penetrate the tubules and form a mineralized layer that may contribute to reducing DH. However, as this study was conducted in a controlled laboratory setting, further research including in vivo and clinical trials is necessary to confirm the long-term effectiveness, safety, and clinical applicability of ZnO NPs in managing DH.

## Data Availability

All data sets and materials employed or examined in the present study are comprehensively documented within this published article.

## Abbreviations

HANPs: Hydroxyapatite Nanoparticles
ZnO NPs: Zinc Oxide Nanoparticles
DTs: Dentinal Tubules
DH: Dentin Hypersensitivity
NPs: Nanoparticles
CMC: Carboxymethyl Cellulose
SEM: Scanning Electron Microscopy
TEM: Transmission Electron Microscopy
EDX: Energy-Dispersive X-ray Spectroscopy
Ca: Calcium
P: Phosphorus
HA: Hydroxyapatite
ZnO: Zinc Oxide
KCl: Potassium Chloride
MgCl2: Magnesium Chloride
H_2_O: Water
CaCl_2_: Calcium Chloride
K_2_HPO_4_: Dipotassium Hydrogen Phosphate
SRa: Average Surface Roughness
FESEM: Field Emission Scanning Electron Microscopy
MMP: Matrix metalloproteinases
Zn: Zinc
ROS: Reactive oxygen species
